# Quartz Crystal Microbalance Humidity Sensors Based on Structured Graphene Oxide Membranes with Magnesium Ions: Design, Mechanism and Performance

**DOI:** 10.3390/membranes12020125

**Published:** 2022-01-21

**Authors:** Ruobing Yi, Bingquan Peng, Yimin Zhao, Dexi Nie, Liang Chen, Lei Zhang

**Affiliations:** 1 MOE Key Laboratory for Nonequilibrium Synthesis and Modulation of Condensed Matter, School of Physics, Xi’an Jiaotong University, Xi’an 710049, China; ruobing94@163.com (R.Y.); zx1959@stu.xjtu.edu.cn (Y.Z.); niedexi19970709@stu.xjtu.edu.cn (D.N.); 2 Wenzhou Institute, University of Chinese Academy of Sciences, Wenzhou 325001, China; pengbq@ucas.ac.cn; 3 Oujiang Laboratory (Zhejiang Lab for Regenerative Medicine, Vision and Brain Health), Wenzhou 325001, China; 4 School of Physical Science and Technology, Ningbo University, Ningbo 315211, China; liang_chen05@126.com

**Keywords:** humidity sensor, graphene oxide, interlayer spacing, response-frequency shift, response/recovery time

## Abstract

The application of graphene oxide (GO)-based membranes combined with a quartz crystal microbalance (QCM) as a humidity sensor has attracted great interest over the past few years. Understanding the influence of the structure of the GO membrane (GOM) on the adsorption/desorption of water molecules and the transport mechanism of water molecules in the membrane is crucial for development of applications using GOM-based humidity sensors. In this paper, by investigating the effects of oxygen-containing groups, flake size and interlayer spacing on the performance of humidity sensing, it was found that humidity-sensing performance could be improved by rational membrane-structure design and the introduction of magnesium ions, which can expand the interlayer spacing. Therefore, a novel HGO&GO&Mg^2+^ structure prepared by uniformly doping magnesium ions into GO&HGO thin composite membranes was designed for humidity sensing from 11.3% RH to 97.3% RH. The corresponding sensor exhibits a greatly improved humidity sensitivity (~34.3 Hz/%RH) compared with the original pure GO-based QCM sensor (~4.0 Hz/%RH). In addition, the sensor exhibits rapid response/recovery times (7 s/6 s), low hysteresis (~3.2%), excellent repeatability and good stability. This research is conducive to understanding the mechanism of GOM-based humidity sensors. Owing to its good humidity-sensing properties, the HGO&GO&Mg^2+^ membrane-based QCM humidity sensor is a good candidate for humidity sensing.

## 1. Introduction

There is an increasing demand for high-precision and sensitive real-time humidity detection, which is important not only for traditional industries, such as agriculture [1], grain storage [2] and industrial production, but also for high-tech industries, such as semiconductor technology, huma-health real-time detection and clean energy storage [3,4]. QCMs have the ability to monitor mass changes at the sub-nanogram level [5,6,7], as well as convert the dynamic-adsorption mass change of thin membranes deposited on electrodes into resonance-frequency-shift information [8,9]. These virtues are required in humidity sensors.

The materials coating a QCM are the key to determining the detection performance of QCM-based humidity sensors [10,11]. Various materials, such as two-dimensional (2D) materials [11,12], polyelectrolytes [13] and metal oxides [14], have been researched. Among them, 2D GO materials have attracted much attention because of their many features, such as a high specific surface area, a high mechanical modulus, easy stacking, easy functionalization and luxuriant oxygen-containing functional groups [15,16,17,18]. However, previous studies [19] found that the interlayer spacing of GOMs is 12.8 ± 0.2 Å, even when completely immersed in water. Considering that interlayer spacing of GOMs at ambient humidity is ~8 Å, this limits the maximum sensitivity of GO-based QCM humidity sensors. In addition, the drawback of easy swelling in the wet state arises from an increase in humidity hysteresis [20,21,22]. Therefore, there have been many efforts in recent years to improve the humidity-sensing performance of GO-based sensors by cross-linking GO with hydrophilic materials, such as polymers [23], metal oxides [14] and nanodiamonds [24].

In this work, considering the complex structure of GO [15,25], it is speculated that the performance of GO-based humidity sensors is affected not only by the hydrophilic strength of the material itself but also the size of the GO flakes, the interlayer spacing, and the self-assembly method of GO flakes. To determine this, the influence of oxygen content and flake size on GO-based humidity-sensing performance was first investigated, and it was found that HGO with a higher oxygen content and smaller sheet size had better humidity sensitivity. GO and HGO were prepared by Hummer’s method at a low temperature and a high temperature [26], respectively. The flake size of GO was larger than that of HGO, while the oxygen content of GO was lower than that of HGO. To further explore the effect of interlayer spacing, magnesium ions were introduced into the GOM and HGOM [19,27]. According to a previous report [19], Mg^2+^ can not only prevent GO from dissolving but also expand the interlayer spacing of GO. Our results show that the introduction of magnesium ions remarkably improved the sensor performance of GO- and HGO-based humidity sensors. In addition, a reasonable composite membrane structure (composed of HGO and GO) can further balance the humidity sensitivity and response/recovery rate. Eventually, the obtained structure (HGO&GO&Mg^2+^) exhibited excellent humidity-sensing properties, showing vast potential for practical applications in the area of humidity sensors.

Humidity-sensing properties, such as sensitivity, repeatability, stability and response/recovery time, were investigated by a QCM. In addition, to understand the mechanism of the high-humidity-sensing performance of HGO&GO&Mg^2+^-based QCM sensors, characterization was performed by atomic force microscopy (AFM), scanning electron microscopy (SEM), energy dispersive X-ray (EDX) spectroscopy, X-ray diffraction (XRD), Fourier transform infrared (FTIR) spectroscopy and X-ray photoelectron spectroscopy (XPS). 

## 2. Materials and Methods

### 2.1. Materials

Graphite powder (325 mesh), sulphuric acid (H_2_SO_4_), potassium persulfate (K_2_S_2_O_8_), phosphorus pentoxide (P_2_O_5_), potassium permanganate (KMnO_4_), hydrogen peroxide (H_2_O_2_), magnesium chloride (MgCl_2_) and hydrochloric acid (HCl) were used in this research. All chemical regents were purchased from Sinopharm Chemical Reagent Co. (Shanghai, China) and used with ultra-pure deionized (DI) water with 18.2 MΩ·cm^−1^ in all experiments. All the reagents were utilized as received, without further purification.

### 2.2. Preparation of GO and HGO Suspension

GO aqueous suspension (5 mg/mL) was prepared according to the modified hummers method, as previously reported [19,28]. Graphite powder was added to concentrated H_2_SO_4_, K_2_S_2_O_8_ and P_2_O_5_ solution and stirred continuously at 80 °C for 4.5 h. The mixture was purified by vacuum filtration and washed with deionized (DI) water. After vacuum drying at 60 °C overnight, oxidized graphite was obtained. The obtained oxidized graphite was further oxidized in concentrated H_2_SO_4_ and KMnO_4_ at constant temperatures of 6 and 60 °C, respectively. The solutions were stirred at 35 °C for 2 h, and then they were diluted with 250 mL DI water at 40 and 80 °C, respectively. The graphene oxide prepared at higher temperatures (oxidized at 60 °C and adding DI water at 80 °C) was named HGO, while the other one was named as GO. The solutions were further stirred at room temperature for 2 h, and H_2_O_2_ (30%, 20 mL) was added immediately after dilution with DI water (700 mL). The product was centrifuged and sequentially washed with a 1:10 aqueous HCl solution and DI water to remove impurities. Finally, the GO and HGO aqueous suspension with a concentration of about 5 mg/mL was prepared. The mixed solution of GO and HGO (GO&HGO) suspension was obtained by mixing equal volumes of HGO and GO solutions with same concentration (5 mg/mL).

### 2.3. Sensor Fabrication

**Figure 1:**
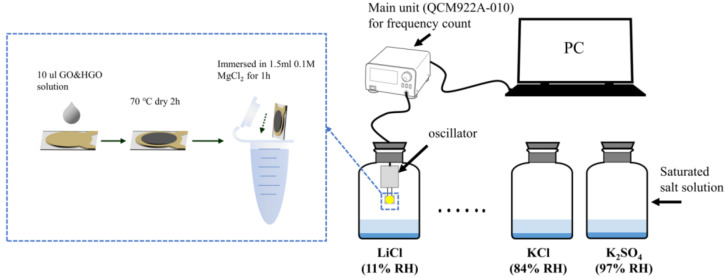
Schematic images of the preparation process of the HGO&GO&Mg^2+^-based quartz crystal microbalance (QCM) sensor and the humidity-sensing experimental setup.

QCM devices (Model 922A, Princeton Applied Research, Oak Ridge, TN, USA) consisting of AT-cut quartz crystals (8 mm diameter) with a fundamental resonance frequency of 9 MHz and Pt electrodes (SEIKO EG&G Co., Ltd., Tokyo, Japan) on both sides were employed in this research. Before the preparation of thin sensing membranes, all QCM devices were washed successively with diluted hydrochloric acid, deionized water and ethanol, and each process was followed by a drying procedure at 70 °C for 30 min. The GO, HGO and GO&HGO sheets were deposited by drop-casting 10 µL of the corresponding suspension with a concentration of 0.1 mg mL^−1^ onto the Pt surface of the QCM electrode and drying at 70 °C for 2 h, as illustrated in Figure 1. The GO (HGO, HGO&GO)-coated Pt electrode was immersed in 1.5 mL of 0.1 M MgCl_2_ at 25 °C for 2 h and was measured after drying at 70 °C for 1 h to improve the adhesion properties of the GOM on the Pt electrode.

### 2.4. Measurement of Relative-Humidity-Sensing Performance and Characterization

The schematic of the experimental system used for relative-humidity (RH)-sensing measurement is illustrated in Figure 1. Measurements were collected at an ambient temperature of 25 °C. Saturated solutions of LiCl, CH_3_COOK, MgCl_2_, K_2_CO_3_, Mg(NO_3_)_2_, NaCl, KCl and K_2_SO_4_ at 25 °C provided 11%, 23%, 33%, 43%, 52%, 75%, 85% and 97% RH levels [29], respectively. The real-time frequency of the QCM sensors under different RH levels was obtained by the QCM and recorded by a computer. During the measurement process, the frequency value (F_0_) of the sensor exposed to 11% RH was used as the baseline to evaluate the performance of the QCM sensors. The recording periods for response and recovery times were fixed at 30 s.

The response-frequency shift was defined as:

(1) $$\Delta F=F-F_{0}$$

where F and F_0_ are the response frequency at the corresponding tested RH level and 11 %RH, respectively. The humidity hysteresis is an important criterion for practical application, defined as:

(2) $$\mathrm{Humidity}\;\mathrm{hysteresis}=\frac{{\Delta F\;}_{\max}\;}{F_{\mathrm{total}}\times 100\%}$$

where ΔF_max_ is the maximum hysteresis error and F_total_ is the total response-frequency-shift output from minimum humidity level (11%) to maximum humidity level (97%).

The FTIR spectra were collected in the mid-infrared range of 4000–1000 cm^−1^ using a Nicolet iS50 FTIR spectrometer (Thermo Fisher Scientific, Shanghai, China), and the XPS results of free-standing GO and HGO membranes were characterized by a Thermo Fisher ESCALAB Xi+ (Thermo Fisher Scientific, Shanghai, China). XRD patterns of membranes were obtained by an X-ray diffractometer system (Bruker D8 Advance, λ = 0.15418 nm; Bruker Corporation, Billerica, MA, USA). XRD spectra of membranes under different relative humidity levels were tested on a glass substrate with a cylindrical groove in the middle. Saturated solutions of different salts were added to the groove, as previously described. These saturated solutions can control the humidity. Meanwhile, the sample was placed on a glass supported by a rubber cement above the liquid level. The whole groove was sealed with plastic wrap during the entire test process. GO and HGO flakes were measured by atomic force microscopy (AFM, SPM-9700HT; Shimadzu Co., Ltd., Tokyo, Japan). SEM images were collected using a Gemini SEM 500 (Carl Zeiss Co., Ltd., Shanghai, China) series field-emission scanning electron microscope operated at an accelerating voltage of 15 kV.

## 3. Results and Discussion

### 3.1. Characterization Results

**Figure 2:**
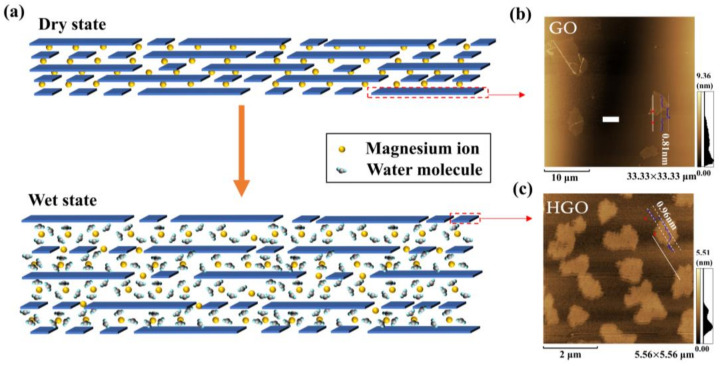
(**a**) Schematic diagram of HGO adsorption/desorption of water molecules. (**b**,**c**) Atomic Force Microscopy (AFM) images of single flakes of Graphene Oxide (GO) and HGO.

Figure 2a shows the humidity-sensing schematic of HGO&GOM&Mg^2+^. It can be clearly seen that the framework is composed of GO and HGO flakes with different lateral sizes. The AFM images in Figure 2b, c provide the details of single flakes of GO and HGO. The results show that the maximum lateral size of GO flakes (approximately 4–10 μm) was approximately 4–10 times larger than that of HGO flakes (~1 μm). Therefore, smaller HGO flakes were fabricated during the process of oxidation and exfoliation at higher reaction temperatures.

**Figure 3:**
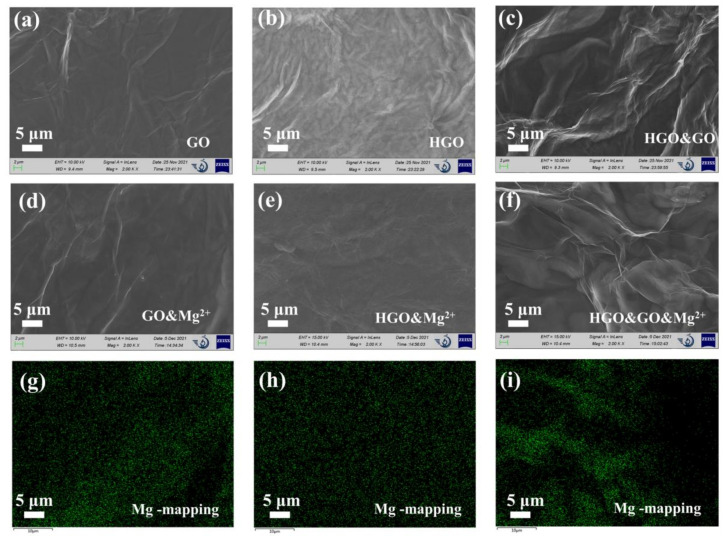
(**a**–**f**) Scanning Electron Microscopy (SEM) images of the GO, HGO, HGO&GO, GO&Mg^2+^, HGO&Mg^2+^ and HGO&GO&Mg^2+^ membranes. (**g**–**i**) Corresponding Mg elemental mapping detected in the energy dispersive X-ray (EDX) spectra of (**d**–**f**), respectively.

The SEM images of GOM, HGOM and HGO&GOM, which are shown in Figure 3a–c, also prove this indirectly. It can be clearly observed that the surfaces of HGOM and HGO&GOM were rougher and more wrinkled than those of GO. It is known that flake size and the oxygen content have a great influence on morphology [28]. As the SEM images show in Figure 3d–f, although the morphologies of GOM&Mg^2+^, HGOM&Mg^2+^ and HGO&GOM&Mg^2+^ are very similar to those of GOM, HGOM and HGO&GOM, it is clear that magnesium was uniformly distributed in these membranes, according to EDX spectroscopy (Figure 3g–i). Moreover, the distribution at the fold was more concentrated.

**Figure 4:**
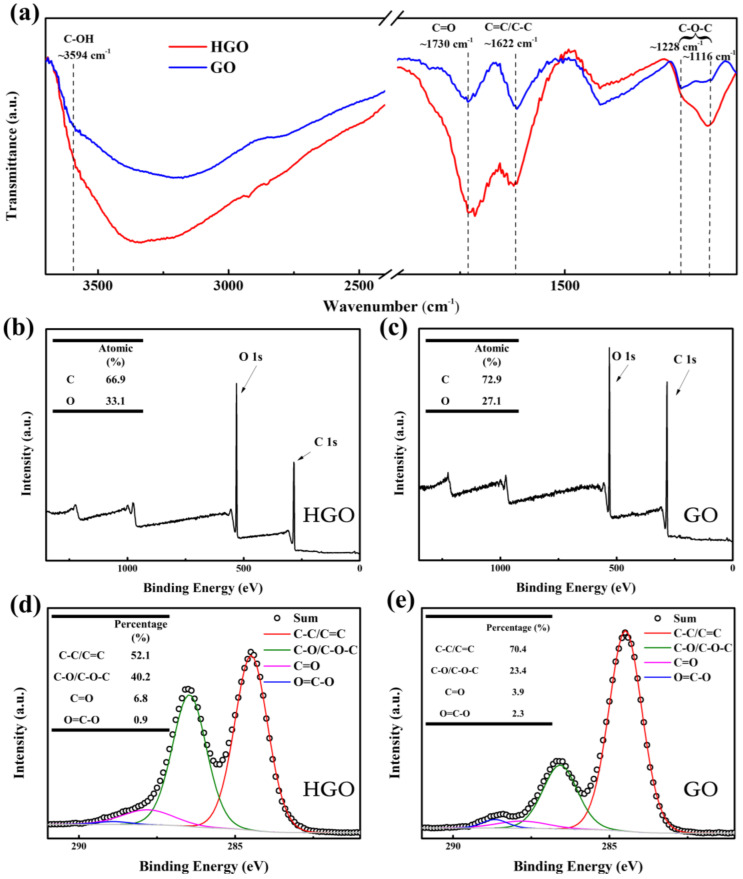
(**a**) The normalized Fourier transform infrared (FTIR) spectroscopy of GO and HGO membranes. (**b**,**c**) The full-scan X-ray photoelectron spectroscopy (XPS) of HGO and GO; inset is the atomic percentage of C and O. (**d**,**e**) C1s XPS spectra of HGO and GO; inset is the proportion of oxygen-containing functional groups.

The number of oxygen-containing groups in GO has a significant impact on the humidity sensitivity of GO-based humidity sensors [11]. To explore this, the FTIR spectra of GO and HGO were detected, as shown in Figure 4a. The main characteristic peaks at ~3594 cm^−1^, 1730 cm^−1^, 1622 cm^−1^ and 1116–1228 cm^−1^ correspond to stretching vibrations of hydroxyl (O–H) groups, carboxyl (C=O) groups, aromatic ring (C–C/C=C) and epoxy (C–O–C) groups [28,30,31], respectively. Obviously, the intensity of the absorption peak after normalization in GO is weaker than that in HGO at 3594 cm^−1^, 1730 cm^−1^, 1622 cm^−1^ and 1116–1228 cm^−1^, which shows that the oxygen content of the former is lower than that of the latter. It can be concluded that a high reaction temperature is beneficial for further intercalation of oxidized groups, which also promotes the exfoliation of graphite oxide into smaller GO sheets. This is identified with the results of AFM. Figure 4b,c show the full scan XPS spectra of HGO and GO. Obviously, the former contains more oxygen than the latter. As shown in Figure 4c,d the proportion of oxygen-containing functional groups can be obtained according to C1s curve-fitting peak analysis. Four peaks at 284.6 eV, 286.7 eV, 287.8 eV and 288.9 eV correspond to C–C/C=C, C–O/C–O–C, C=O and O–C=O, respectively [3,19,32,33]. It can be observed that the amount of hydroxyl/epoxy in GO is lower than that in HGO, which is consistent with the FTIR results.

**Figure 5:**
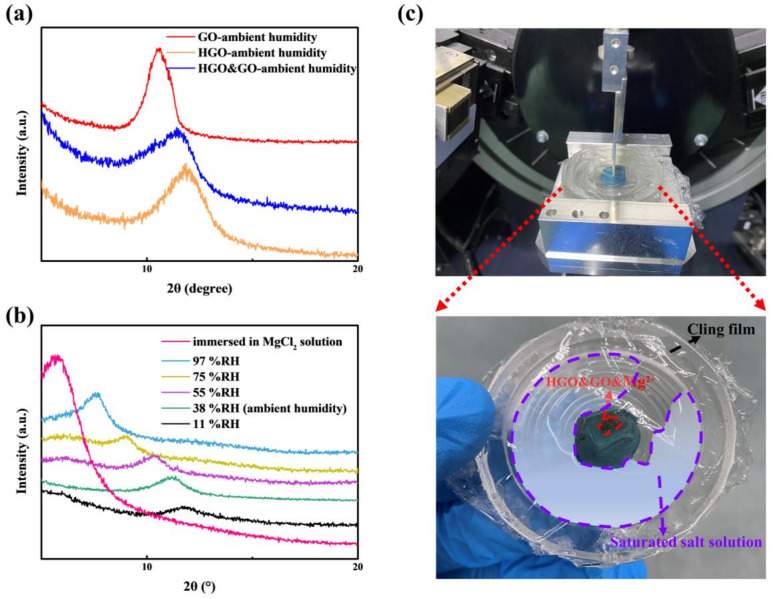
(**a**) X-ray diffraction (XRD) spectroscopy of dry GO, HGO and HGO&GO membranes. (**b**) XRD spectra of HGO&GO&Mg^2+^ membranes under different relative humidity levels and HGO&GO membranes immersed in MgCl_2_ solution. (**c**) Images of the substrate for controlling humidity used in XRD detection.

It is easily understood that the interlayer spacing of GOMs may have a direct influence on their ability to adsorb water molecules [10]. As shown in Figure 5a, the interlayer spacing of GO (~8.3 Å) is greater than that of HGO (~7.5 Å) at ambient humidity, while that of HGO&GO (~7.8 Å) is between those of HGO and GO. Somewhat curiously, the interlayer spacing of HGO with higher oxygen content is less than the interlayer spacing of the lower-oxygen-content GO, probably because the stacking of smaller HGO flakes is more compact than that of the GO flakes during the self-assembly process. As shown in Figure 5b, the interlayer spacings of HGO&GO&Mg^2+^ under different humidity levels (11%, 38%, 55%, 75% and 97% RH) are 7.5 Å, 7.9 Å, 8.5 Å, 9.7 Å and 11.7 Å, respectively. It can be easily observed that the interlayer spacing of HGO&GO&Mg^2+^ increases with increasing humidity. The interlayer spacing at 97% RH is ~4.2 Å greater than that at 11% RH, while the interlayer spacing of HGO&GOM completely immersed in MgCl_2_ solution is 15.3 Å. According to a previous report, the interlayer spacing of GO controlled by MgCl_2_ solution is 13.6 Å, which is less than that of the HGO&GO controlled by MgCl_2_ solution obtained in this work. From the full-width half maximum (FWHM) of the characteristic peak, MgCl_2_-solution-controlled GOM exhibits a greater degree of lamellar orientation, which is consistent with our previous report [19]. Since the XRD test under controlled humidity conditions requires a layer of cling film (to keep enclosure space, as shown in Figure 5c on the surface of the HGO&GO membranes, it can be seen in Figure 5b that all the signals are weak and have a large FWHM, except for the XRD peak of MgCl_2_-solution-immersed HGO&GOM. In summary, HGO&GO&Mg^2+^ possesses a large interlayer spacing and a greater degree of lamellar orientation, which may be beneficial for the adsorption capacity of water molecules and the transport rate of water molecules inside the membrane. 

### 3.2. Humidity-Sensing Properties

**Figure 6:**
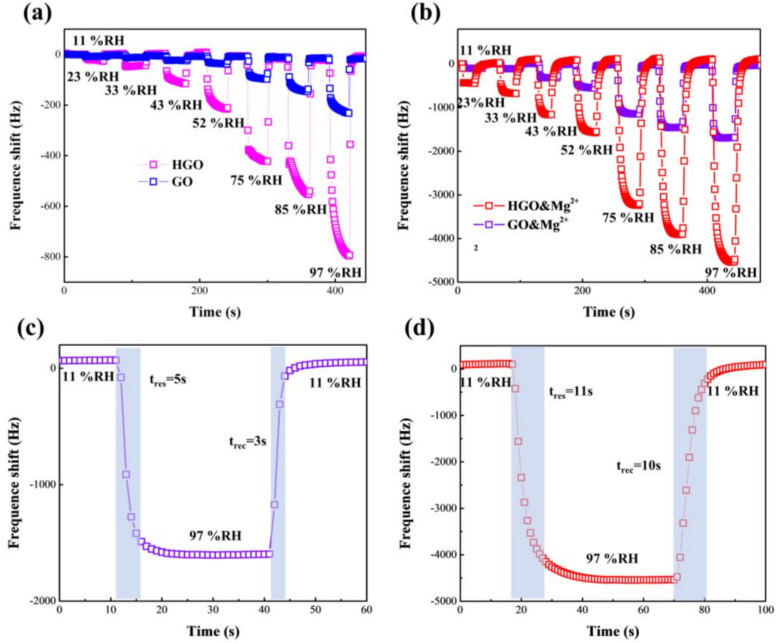
(**a**) Response-frequency shift of HGO- and GO-based QCM humidity sensors with relative-humidity (RH) of 11% to 97%. (**b**) Response-frequency shift of HGO&Mg^2+^- and GO&Mg^2+^-based QCM humidity sensors with RH of 11% to 97%. (**c**,**d**) Response and recovery times of HGO&Mg^2+^- and GO&Mg^2+^-based QCM humidity sensors, respectively, switching from 11% RH to 97% RH.

The response-frequency shifts (ΔF) of the GO- and HGO-based sensors with increasing RH levels in the range of 11–97% RH are shown in Figure 6a,b. It can be clearly found that the response, ΔF, of the two sensors both increase with increasing humidity. The maximum response, ΔF, of HGO- and GO-based sensors were 232 Hz and 793 Hz, respectively, in the relative humidity range from 11 to 97% RH. The response, ΔF, of the HGO-based sensor was greater than that of the GO-based sensor, suggesting that a smaller flake size and higher oxygen content are conducive to absorbing water molecules. This performance is consistent with that observed in previous works [34]. Moreover, the GO&Mg^2+^- and HGO&Mg^2+^-based sensors both exhibit significant strengthening in response, ΔF, compared with the GO- and HGO-based sensors. Although the response, ΔF, of the GO&Mg^2+^-based sensor (19.6 Hz/%RH) is not as high as that of the HGO&Mg^2+^-based sensor (52.7 Hz/%RH), the former still increased to approximately 10 times that of the original GO-based sensor. Because the size of GO flakes is too large and the oxygen-content is low, it is difficult for water molecules to enter the deep area when GO is not completely wet. Therefore, the interior of GO membranes may not be fully utilized. The introduction of Mg^2+^ increases the interlayer spacing of the GO and makes it easier for water to enter, while this has a relatively smaller effect on HGO. This is in agreement with the XRD results. In addition, the evenly distributed Mg^2+^ ions have a certain degree of hydrophilicity [35,36,37], which is also beneficial for improvement in the response, ΔF.

To assess the dynamic response performance of the sensors, the response and recovery times between 11% RH and 97% RH were also measured. Response and recovery time (t_res_ and t_rec_) in this work is defined as the time for the response, ΔF, to achieve 90% of the total ΔF amount between two different humidity levels, respectively, during the process of adsorption and desorption. As shown in Figure 6c,d, the GO&Mg^2+^-based sensor presents a shorter t_res_/t_rec_ (5 s/3 s) than the HGO&Mg^2+^-based sensor (11 s/10 s). The reason is that on one hand, the response, ΔF, of GO is relatively smaller, and on the other hand, the large area of sp^2^ hydrophobic channels in the GO membrane can help oxygen-containing groups and Mg^2+^ ions more quickly absorb and desorb water molecules [38,39,40]. Considering that the combination of HGO with Mg^2+^ can greatly improve the response-frequency shift and GO with Mg^2+^ can decrease response/recovery time of the QCM sensor, respectively, a more practical sensor may be prepared by combining HGO, GO and Mg^2+^.

**Figure 7:**
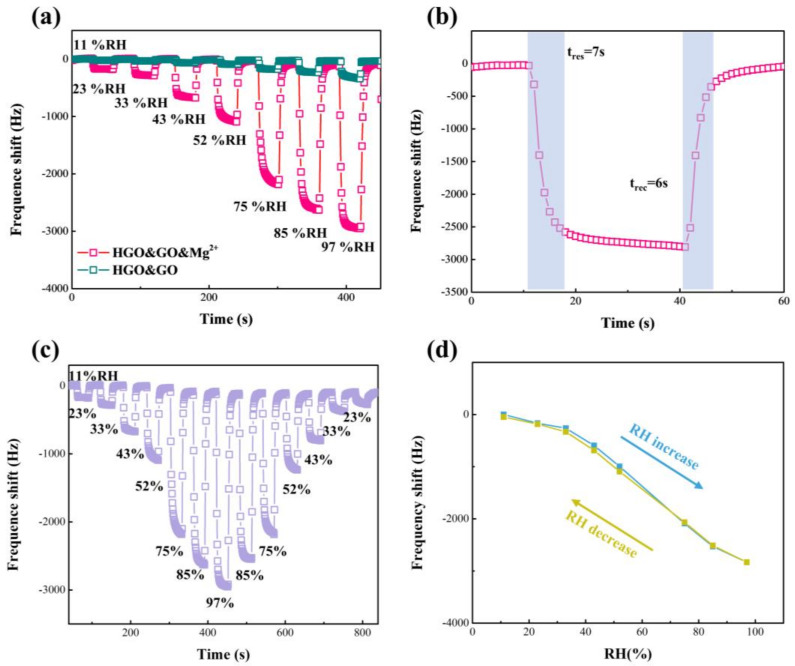
(**a**) Response-frequency shift of HGO&GO- and HGO&GO&Mg^2+^-based QCM humidity sensors with RH of 11% to 97%. (**b**) Response-frequency shift of HGO&GO&Mg^2+^-based QCM humidity sensors with RH of 11% to 97%. The dynamic humidity hysteresis loop (**c**) and the humidity hysteresis curve (**d**) versus time for HGO&GO&Mg^2+^-based QCM humidity sensors with RH of 11% to 97%.

The response, ΔF, humidity hysteresis and t_res_/t_rec_ of the HGO&GO&Mg^2+^-based sensor were further measured to evaluate its humidity-sensing performance, as shown in Figure 7. It is easily observed that the performance of the HGO&GO&Mg^2+^-based sensor shows a remarkable strengthening compared with the HGO&GO-based sensor, suggesting that MgCl_2_ plays an important role in improving the humidity-sensing ability. The response, ΔF, of the HGO&GO&Mg^2+^-based sensor (34.3 Hz/%RH) decreased compared to that of HGO&Mg^2+^ (52.7 Hz/%RH), while the t_res_ and t_rec_ of the former were reduced to 7 s and 6 s, respectively. Figure 7c shows the dynamic response, ΔF, of the humidification process (from 11% RH to 97% RH), corresponding to the absorption of water molecules, and the dehumidification process (from 97% RH to 11% RH), corresponding to the desorption of water molecules. The difference between the two processes is shown in Figure 7d. The maximum ΔF difference between the absorption and desorption processes is only 96 Hz, which corresponds to a humidity hysteresis of only ~3.2 RH%. The humidity hysteresis is calculated according to Equations (1) and (2). The reason for such a low humidity hysteresis is that Mg^2+^ can effectively limit the swelling of GO [19]. These results suggest good reversibility between the humidification and dehumidification processes.

Table 1 shows the comparison of humidity sensing between the GO-based humidity sensors obtained in this work and previous works. “@90% RH (1–97%RH)” means the humidity range for testing reaction/recovery times in this work ranges from 11 to 97%, and the response and recovery times are defined as the time for the response, ΔF, to achieve 90% of the total amount during the processes of adsorption and desorption, respectively. In this situation, it is apparent that the HGO&GO&Mg^2+^-based QCM sensor is better than most GO-based QCM sensors not only in terms of sensitivity but also in response/recovery time. The HGO&GO&Mg^2+^-based QCM sensor is also excellent, even compared to other materials used for humidity sensors.

**Figure 8:**
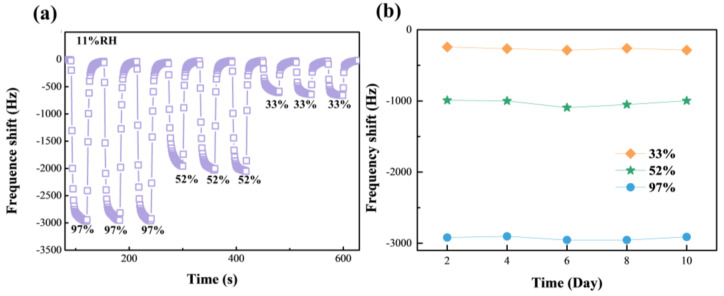
The repeatability (**a**) and stability (**b**) test of the HGO&GO&Mg^2+^-based QCM humidity sensor.

Stability and repeatability are two important criteria to measure the practical value of humidity-sensor performance. Figure 8a shows the repeatability of the HGO&GO&Mg^2+^-based sensor for different humidity levels: 11% to 33% RH and 52% to 97% RH. There is no clear variation in the response, ΔF, under the same humidity conversion conditions, suggesting that the HGO&GO&Mg^2+^-based sensor has excellent repeatability. In addition, as observed in Figure 8b, the response, ΔF of the HGO&GO&Mg^2+^-based sensor at three humidity levels (33% RH, 52% RH and 97% RH) was tested every two days for 10 days, and a minimal variation in the response, ΔF, appeared at different humidity levels. This obviously means that the HGO&GO&Mg^2+^-based sensor has good stability.

### 3.3. The Mechanism of Humidity Sensing

According to the aforementioned experimental results, the humidity-sensing performance of the HGO&GO&Mg^2+^-based sensor represents a remarkable enhancement compared with that of GO and HGO. Previous work [20] has reported the interlayer spacing of GOM under relative humidity from 0% to 100%. Interlayer spacing enlarges with increased humidity. However, even if the RH reaches 100%, the interlayer spacing of GO is approximately 3 Å larger than that in the case of 0% RH. Compared with the XRD results in this work, the introduction of Mg^2+^ can further enlarge the interlayer spacing of HGO&GOM in different humidity states. Moreover, the interlayer spacing at 97% RH is ~4.2 Å larger than that at 11% RH, which means that HGO&GO&Mg^2+^ can attract and hold more water molecules. Compared with that of GO or HGO, the reason why the humidity-sensing performance of the HGO&GO&Mg^2+^-based sensor represents a remarkable enhancement can be summarized by four aspects:

1. The introduction of magnesium ions can increase the interlayer spacing of GOMs, which is beneficial for attracting and holding more water molecules. This can be proven by XRD results.
2. Mg^2+^ can prevent the swelling of GOMs [19], which is helpful for decreasing humidity hysteresis.
3. Mg^2+^ has a certain degree of water-absorption capacity [35,36,37]. More water molecules can be introduced into GOMs by evenly distributed magnesium ions. It is believed that ions with similar properties to magnesium ions can also increase the humidity-sensing performance of GO films.
4. For the composite structure of GO and HGO, on one hand, a large number of oxygen-containing groups in HGO provides many adsorption sites for water molecules; on the other hand, a large number of sp^2^ hydrophobic regions in the large layer of GO is conducive to the rapid transmission of water molecules inside the membrane. These are both conducive to improving humidity-sensing performance. In short, a mixed structure of GO and HGO has the ability to balance response sensitivity and response/recovery time.

## 4. Conclusions

In summary, the humidity-sensing properties of GO-based humidity sensors can be significantly enhanced by introducing Mg^2+^ and a reasonable structural design. The obtained HGO&GO&Mg^2+^ sensor exhibits a high response-frequency shift (34.3 Hz/%RH), short response/recovery times (7 s/6 s), low humidity hysteresis (3.2%) from 11% RH to 97% RH and good repeatability/stability. These two improved strategies are based on the fact that Mg^2+^ not only expands the interlayer spacing of GOMs but also attracts more water molecules into the interior of GOMs, while the unique structure composed of HGO and GO still ensures a rapid response/recovery time when humidity sensitivity is vastly improved. Most importantly, the findings in this work contribute to further understanding of the transport mechanism of water molecules in GOMs, which promotes the application of not only QCM humidity materials but also fuel-cell, water-desalination and nanofluidic devices.

## Figures and Tables

**Table 1:** Comparison between recently reported QCM humidity sensors and this work.

Materials	Sensing Range (%RH)	Sensitivity (Hz/%RH)	Response/Recover Time (s)	Published Year	Ref
RGO-PEO	0–84	20	11 s/7 s@63.2% RH (0–84% RH)	2018	[41]
PANI/GO	0–97	20	8 s/5 s@63.2% RH (0–97% RH)	2018	[13]
GO/C_60_	11–97	31	72 s/8 s@90% RH (0–97% RH)	2018	[42]
GO/SnO_2_/PANI	0–97	29.1	7 s/2 s@63.2% RH (0–97% RH)	2018	[43]
GO	10–90	5.6	not given	2020	[34]
GO	10–70	10.9	not given	2019	[44]
MoS_2_/GO/C_60_-OH	2–97	31.8	1.3 s/1.2s@90% RH (54–97% RH)	2021	[10]
PAN/PEI	38–78	154.5	13 s/7 s@90% RH (38–78% RH)	2020	[45]
MXene nanosheets	11–97	12.8	6 s/2 s@90% RH (11–97% RH)	2021	[46]
PPy/CS	0–97	52.9	13 s/2 s@90% RH (0–97% RH)	2021	[47]
GO&Mg^2+^	11–97	19.6	5 s/3 s@90% RH (11–97% RH)	2021	This work
HGO&Mg^2+^	11–97	52.7	11 s/10 s@90% RH (11–97% RH)	2021	This work
HGO&GO&Mg^2+^	11–97	34.3	7 s/6 s@90% RH (11–97% RH)	2021	This work

“@*x* % RH (*y*–*z*% RH)” means the humidity range for testing reaction/recovery times in this work from *y* to *z* % RH. The response and recovery times are defined as the time for the response, ΔF, to achieve *x* % of the total ΔF amount between two different humidity levels during the processes of adsorption and desorption, respectively.

## Data Availability

The data presented in this study are available on request from the corresponding author.
